# Validation of microbial source tracking markers for the attribution of fecal contamination in indoor-household environments of the Peruvian Amazon

**DOI:** 10.1016/j.scitotenv.2020.140531

**Published:** 2020-11-15

**Authors:** Francesca Schiaffino, Nora Pisanic, Josh M. Colston, Dixner Rengifo, Maribel Paredes Olortegui, Valentino Shapiama, Pablo Peñataro Yori, Christopher D. Heaney, Meghan F. Davis, Margaret N. Kosek

**Affiliations:** a Department of International Health, Johns Hopkins Bloomberg School of Public Health, Baltimore, MD, USA; b Faculty of Veterinary Medicine, Universidad Peruana Cayetano Heredia, San Martin de Porres, Lima, Peru; c Department of Environmental Health and Engineering, Johns Hopkins Bloomberg School of Public Health, Baltimore, MD, USA; d Division of Infectious Diseases, University of Virginia, Charlottesville, VA, USA; e Asociacion Benefica Prisma, Iquitos, Peru; f Department of Epidemiology, Johns Hopkins Bloomberg School of Public Health, Baltimore, MD, USA; g Department of Molecular and Comparative Pathobiology, Johns Hopkins Bloomberg School of Medicine, Baltimore, MD, USA

**Keywords:** Microbial source tracking, qPCR, Feces, Validation

## Abstract

The performance of eight microbial source tracking (MST) markers was evaluated in a low-resource, tropical community located in Iquitos, Peru. Fecal samples from humans, dogs, cats, rats, goats, buffalos, guinea-pigs, chickens, ducks, pigeons, and parrots were collected (*n* = 117). All samples were tested with human (*BacHum, HF183-Taqman*), dog (*BactCan*), pig (*Pig-2-Bac*), and avian (*LA35, Av4143, ND5, cytB*) markers using quantitative PCR (qPCR). Internal validity metrics were calculated using all animal fecal samples, as well as animal fecal samples contextually relevant for the Peruvian Amazon. Overall, *Pig-2-Bac* performed best, with 100% sensitivity and 88.5% specificity to detect the correct fecal source. Human-associated markers showed a sensitivity of 80.0% and 76.7%, and specificity of 66.2% and 67.6%. When limiting the analysis to contextually relevant animal fecal samples for the Peruvian Amazon, *Av143* surpassed *cytB* with 95.7% sensitivity and 81.8% specificity. *BactCan* demonstrated 100% sensitivity and 47.4% specificity. The gene copy number detected by *BacHum* and *HF183-Taqman* were positively correlated (Pearson's correlation coefficient: 0.785), as well as avian markers *cytB* with *Av4143* (Pearson's correlation coefficient: 0.508) and *nd5* (Pearson's correlation coefficient: 0.949). These findings suggest that markers such as Av4143, Pig2Bac, cytb and *BacHum* have acceptable performance to be impactful in source attribution studies for zoonotic enteric disease transmission in this and similar low-resource communities.

## Highlights

• First report of six fecal microbial source tracking markers and two avian mitochondrial microbial source tracking markers in the Peruvian Amazon.
• *Pig-2-Bac* was the best performing marker, with 100% sensitivity and 88.5% specificity.
• Based on the sensitivity and specificity, it is possible to use *Av413, cytb and HF183-Taqman* markers for the attribution of fecal contamination and exposure in this setting.

## Graphical abstract

**Figure:**
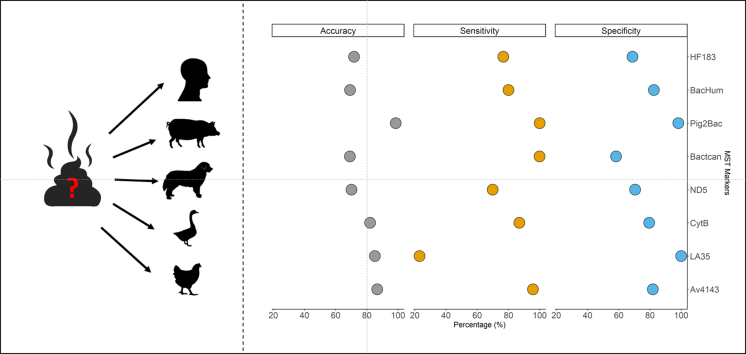

## 1 Introduction

Fecal contamination and associated exposure to enteric pathogens is commonly recognized within the domestic domain in settings in communities living in poverty (Pickering et al., 2012). Traditional water, sanitation and hygiene interventions aim to reduce human fecal contamination of the environment, especially soils and other surfaces that are in routine close contact with children and other household members. However, identification and quantification of animal fecal burden in the household environment has garnered only limited attention and risk assessment to date (Berendes et al., 2018; Penakalapati et al., 2017; Matilla et al., 2018). Zoonotic enteric pathogens, including *Campylobacter* spp., *Cryptosporidium* sp. and Shiga (Vero) toxin producing *Escherichia coli* (STEC) are responsible for a significant proportion of diarrhea-attributed deaths and disease burden (Penakalapati et al., 2017; Collaborators GBDDD, 2017). As a result, not taking into account the role of animal fecal waste in observational and intervention studies that aim to reduce diarrheal incidence in pediatric populations of the developing world may fail to detect important sources of enteric disease burden. In fact, recent water sanitation and hygiene trials in low income settings that manage human fecal waste alone as a mechanism for reducing childhood diarrhea have proven inconsequential, and certain authors have suggested that sources of human pathogens likely extend beyond human fecal exposure to include animal sources (Cumming et al., 2019; Pickering et al., 2019; Luby et al., 2018; Null et al., 2018; Humphrey et al., 2019; Prendergast et al., 2019).

Determination and quantification of fecal contamination using traditional fecal indicator bacteria such as *Escherichia coli* and *Enteroccoccus* has been applied despite significant assay shortcomings (Exum et al., 2016). These indicator bacteria multiply in tropical climates, so detected amounts do not in all cases directly reflect initial contamination levels. Furthermore, these traditional microbiologic methods do not identify the source of fecal contamination; thus, in settings where multiple exposures exist, it is impossible to assign risk to particular competing sources of fecal exposure in order to inform interventions. As a result, there is a need to develop and implement microbiologic tools that enable us to allocate and accurately quantify fecal contamination and attribute the contamination to specific animal species.

Microbial source tracking (MST) has been developed as a tool to quantify and allocate the source of fecal contamination in water to animal sources at the species level. *Bacteroidales* are strictly anaerobic commensal bacteria of the human and animal gut that account for a large percentage of the human gastrointestinal flora (Sghir et al., 2000; Hold et al., 2002). These bacteria have been extensively used as a MST tool given the bacterium's ability to adapt to the intestinal environment of specific animal hosts (Dick et al., 2005). Other markers targeting host-specific enteric bacterial flora, as well as mitochondrial DNA segments, have also been developed (Kildare et al., 2007; Bernhard and Field, 2000; Boehm et al., 2013; Weidhaas et al., 2010; Ohad et al., 2016; Zhuang et al., 2017). Validation of MST markers has proven that their discriminatory power is site specific, and as a result, requires a performance evaluation to determine the sensitivity, specificity and accuracy of each marker (Gawler et al., 2007). This study validates eight MST markers for the determination and quantification of human and animal exposures in the Peruvian Amazon to inform their future implementation in risk assessment measures of animal fecal contamination in this and similar domestic settings in low-income, tropical communities. We focus on the indoor household environment, including household surface samples, for future evaluation of water, hygiene and sanitation trials that aim to reduce enteropathogen transmission (Prendergast et al., 2019).

## 2 Methods

### 2.1 Study setting

Between July and August of 2018, fecal samples used in the validation procedures were collected in Santa Clara de Nanay and Santo Tomas, two peri-urban communities each with a population of 5000 individuals located near Iquitos, Loreto, the largest city in the Peruvian Amazon.

### 2.2 Microbial source tracking markers

Eight MST markers were selected for validation. Four markers targeted avian species (*LA35*, *Av4143*, *ND5* and *CytB*) and the other four targeted mammalian species including humans (*HF183-Taq* and *BacHum*), dogs (*BacCan*) and pigs (*Pig2Bac*)(Kildare et al., 2007; Weidhaas et al., 2010; Ohad et al., 2016; Zhuang et al., 2017; Pisanic et al., 2015). Two markers (*cytB* and *ND5*) targeted avian mitochondrial DNA segments, and the remaining six targeted host-specific gastrointestinal bacteria, including *Brevibacterium avium (LA35)*, a species *of Lactobacillus* spp. (*Av4143*) and host-specific *Bacteroidales* (Table 1).

**Table 1:** Characteristics, primers, probes and origin of microbial source tracking (MST) markers validates in Iquitos, Peru.

Host	Target	Marker	Primers and probes		Reported sensitivity	Reported specificity	References
Chickens	*Brevibacterium avium*	LA35	LA35F	5′-ACC GGA TAC GAC CAT CTG C-3′	60–76%	100%	(Weidhaas et al., 2010; Weidhaas and Lipscomb, 2013)
			LA35R	5′-TCC CCA GTG TCA GTC ACA GC-3′			
			Probe	5′-FAM-CAG CAG GGA AGA AGC CTT CGG GTG ACG GTA-BHQ1-3′			
Chickens and Ducks	Mitochondrial DNA (NADH dehydrogenase subunit 5)	ND5	ND5-F	5′-ACCTCCCCCAACTAGC-3′	100%	84.60%	(Zhuang et al., 2017)
			ND5-R	5′-TTGCCAATGGTTAGGCAGGAG-3′			
			ND5-P	5′-FAM-TCAACCCATGCCTTCTT-NFQ-MGB-3′			
Chickens and Ducks	Mitochondrial DNA (cytochrome *b*)	CytB	cytb-F	5′-AAATCCCACCCCCTACTAAAAATAAT-3′	100%	89.80%	(Zhuang et al., 2017)
			cytb-R	5′-CAGATGAAGAAGAATGAGGCG-3′			
			cytb-P	5′-FAM-ACAACTCCCTAATCGACCT-NFQ-MGB-3′			
Domestic Birds and Waterfowl	*Lactobacillus* spp.	Av4143	Av4143F	5′-TGCAAGTCGAACGAGGATTTCT-3′	95%	97%	(Ohad et al., 2016)
			Av4143R	5′-TCACCTTGGTAGGCCGTTACC-3′			
			Av4143P	5′-FAM-AGGTGGTTTTGCTATCGCTTT-BHQplus-3′			
Dogs	*Bacteroidales*	BacCan	BactCan545f1	5′-GGAGCGCAGACGGGTTTT-3′	57–63%	90–96%	(Kildare et al., 2007; Ryu et al., 2014)
			Uni/Cow690r1	5′-CAATCGGAGTTCTTCGTGATATCTA-3′			
			Uni/Cow 690r2	5′-AATCGGAGTTCCTCGTGATATCTA-3′			
			Uni/Cow 656p	5′-FAM-TGGTGTAGCGGTGAAA-MGB-3′			
Pigs	*Bacteroidales*	Pig2Bac	Pig-2-Bac41F	5′-GCATGAATTTAGCTTGCTAAATTTGAT-3′	100%	100%	(Ryu et al., 2014)
			Pig-2-Bac163Rv	5′-ACCTCATACGGTATTAATCCGC-3′			
			Pig-2Bac113	5′-VIC-TCCACGGGATAGCC-NFQ-MGB-3′			
Humans	*Bacteroidales*	*HF183*-Taq	*HF183*f	5′-ATCATGAGTTCACATGTCCG-3′	29–100%	80–87%	(Kildare et al., 2007; Odagiri et al., 2015)
			BthetR1	5′-CGTAGGAGTTTGGACCGTGT-3′			
			BthetP1	5′-FAM-CTGAGAGGAAGGTCCCCCACATTGGA-TAMRA-3′			
Humans	*Bacteroidales*	*BacHum*	*BacHum*160Fw	5′-TGAGTTCACATGTCCGCATGA-3′	100%	87%	(Kildare et al., 2007)
			*BacHum*241Rv	5′-CGTTACCCCGCCTACTATCTAATG-3′			
			*BacHum*193Probe	5′-FAM-TCCGGTAGACGATGGGGATGCGTT-TAMRA-3′			

### 2.3 Fecal sample collection

Animal fecal samples (*n* = 117) were collected in sterile 2 mL tubes and stored at -20 °C until DNA extraction. Each specimen was from a single individual. Dog (*Canis lupus familiaris*) (*n* = 10), chicken (*Gallus gallus*) (*n* = 13), duck (*Anas platyrhynchos*) (n = 10), parakeet (*Brotogeris versicolurus*) (*n* = 2), and pig (*Sus domesticus*) (n = 10) samples were collected from domestic animals of local households. Guinea-pig (*Cavia porcellus*) (*n* = 5), buffalo (*Bubabuls bubalis*) (n = 5) and goat (*Capra aegagrus hircus*) (n = 10) fecal samples were collected from one single farm in Santo Tomas. Pigeon (*Columba livia*) (n = 10) samples were collected from Iquitos city center. Animals were not touched during sample collection and feces were collected, as soon as the animal defecated, without touching the ground. Cat (*Felis catus*) (n = 2) samples were donated by a local veterinarian. Rat (*Rattus* sp.) (n = 10) and human (healthy children (*n* = 15) and adults (n = 15)) were obtained from the Kosek-Yori biorepository in Iquitos. Demographic characteristics of human fecal samples are shown in the Supplementary Table 1. Based on a previous community census on animal ownership, only dogs, cats, chickens, ducks, pigs and parrots were found in more than 5% of households. These animals will be referred to as “*relevant*” animals throughout the paper. Guinea pigs and goats are seldom found in these communities. Rats were not included in the census but are known to be commonly found within and around households. All MST markers, except *LA35*, were able to detect higher concentrations of gene copy numbers (GCN) in target animal fecal samples in comparison to non-target animal species.

### 2.4 Sample processing and validation assays

DNA was extracted from 0.10 g of feces using PowerSoil® DNA extraction kit (Qiagen, Germantown, MD, USA) following beadbeating according to the manufacturer's instructions. For each extraction, a negative control consisting of RNA free water was used. TaqMan assays consisted of final reaction mixtures of 20uL, which included TaqMan™ Advanced Fast Start Master Mix (2×) (Applied Biosystems, Foster City, CA), forward and reverse primers (200uM), probes (100uM), 5uL of DNA template and RNA free-water (Ambion™, Thermo Fisher Scientific, Waltham, MA, USA). Negative template controls (RNAse free water) were included in each amplification reaction. Reaction mixtures were placed in a 96-well plate and amplified using a StepOnePlus real time PCR system (Applied Biosystems, Foster City, CA). Internal amplification controls (qHsaCtlP0001003, Bio-Rad Laboratories, Irvine, CA) to determine qPCR inhibition were run for every marker and fecal sample and runs were considered invalid if the internal amplification control was above the cycle threshold of 35. Standard amplification conditions (95 °C for 5 min, 40 cycles of 95 °C for 15 s, 53 °C for 15 s, 60 °C for 45 s) were used for all reactions, except for *LA35* and *Av4143*, for which annealing temperatures were set at 56 °C and 55 °C respectively.

### 2.5 Standard curve analysis

MST markers were validated by assessing cross reactivity of each marker with target and non-target animal and/or human fecal samples through quantitative polymerase chain reaction (qPCR) amplification. Standard curves were prepared using 10-fold serial dilutions (3.0 × 10^5^–3.0 × 10^0^ gene copies/uL) of double-stranded synthetic DNA fragments (gBlocks®, Integrated DNA Technologies, Coralville, IA, USA) custom-manufactured for each specific marker. The specific sequences of each gBlock are found in Supplementary Table 2. In order to prepare the working solution of the gBlock, the amount (fmoles) delivered in each control was diluted in 250uL of RNAse free water (Ambion™, Thermo Fisher Scientific, Waltham, MA, USA). The molar concentration (mol/L) and gene copy concentration (copies/uL) of the stock solution were calculated. Ten-fold serial dilutions were prepared by diluting the stock solution with 10 mM Tris-HCL (Quality Bological INC, Gaithersburg, MD, USA) + 0.05% Tween20 (Thermo Fisher Scientific, Waltham, MA, USA). DNA from fecal material was diluted 10-fold diluted and tested in duplicate.

For each specific MST assay, the lower limit of quantification (LLOQ) was set as the average cycle threshold value corresponding to the lowest concentration within the linear range of quantification where at least 95% of the dilution repetitions were detected. The limit of detection (LOD) was set as the LLOQ rounded to the nearest whole number. Additional standard curve parameters included percent efficiency, calculated as: −1 * 10^(−1/slope)^), the slope of the curve and the y-intercept. A sample was considered positive if the Ct value obtained was within the range of quantification. A sample was considered negative if the Ct value obtained was outside the range of quantification or if the target was not detected. If results from a duplicate run where incongruent with each other, the sample was run for a third time and a positive or negative result was assigned based on the result of the results.

### 2.6 Statistical analysis

Internal validity metrics, including sensitivity, specificity, positive predictive value, negative predictive value, and accuracy of each MST marker was calculated according to the species for which each was developed.. Gene quantities were normalized by log(10)-transforming all values. The abundance of gene copy numbers in target and non-target samples was compared using a one-way ANOVA. Pearson's correlation coefficients were estimated for gene copy abundances of all microbial source tracking markers tested, with Bonferroni-adjusted significance levels of 0.05. Data handling and statistical analysis were performed in STATA 14 (Stata Corp., College Station, TX) and R (Version 3.3.2).

## 3 Results

**Fig. 1:**
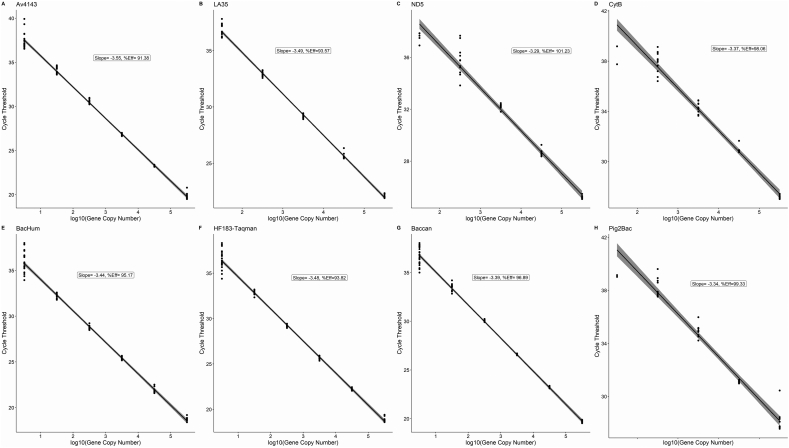
Standard curves of eight microbial source tracking markers validated with fecal samples from Iquitos, Peru.

Standard curves for each microbial source marker tested are presented in Fig. 1. Associated parameters for each curve, including the slope, y-intercept, efficiency (%), lower limit of quantification (LLOQ) and assay specific limit of detection (LOD) is presented in Table 2. Amplification efficiencies ranged between 91.3% and 101.2%. The lower limit of quantification was 3 gene copy numbers/uL for Av4143, *BactCan* and *HF183*, 30 gene copy numbers/uL for ND5, and LA35, and 300 copy numbers/uL for *CytB* and Pig2Bac.

**Table 2:** Standard curve parameters of eight microbial source tracking markers standardized in this study.

Target Specie	MST Marker	Slope	y-intercept	Efficiency (%)	LLOQ (Ct)	LLOQ (gene copy number/uL)	Assay LOD
Domestic Birds and Waterfowl	*Av4143*	−3.55	39.22	91.38	37.42	3	37
Chickens	*LA35*	−3.49	41.32	93.57	36.80	30	37
Chickens and Ducks	*ND5*	−3.29	43.44	101.23	37.55	30	37
Chickens and Ducks	*CytB*	−3.37	45.88	98.06	37.94	300	37
Humans	*HF183Taq*	−3.48	37.83	93.82	36.45	3	36
Humans	*BacHum*	−3.44	37.40	95.17	35.86	3	36
Pigs	*Pig2Bac*	−3.34	45.99	99.33	38.16	300	38
Dogs	*BacCan*	−3.39	38.39	96.89	36.67	3	37

Key: Slope = y-intercept of the curve. LLOQ = lower limit of quantification: average cycle threshold value corresponding to the lowest concentration within the linear range of quantification where at least 95% of the dilution repetitions were detected. LOS = limit of detection: LLOQ rounded to the nearest whole number. Efficiency = (−1 * 10^(−1/slope)).

Internal validity metrics of all eight microbial source tracking markers are shown in Table 3 and associated individual sample results used to calculate these parameters is shown in Table 4. Four avian markers were tested: two (*ND5* and *cytb*) targeting avian mitochondrial gene segments of chickens and ducks, one targeting a gene segment of *Lactobacillus* sp. associated with domestic and waterfowl birds (*Av4143*) and one targeting a gene segment of *Brevibacterium avium* associated with chickens only (*LA35*) (Ohad et al., 2016; Zhuang et al., 2017; Weidhaas and Lipscomb, 2013). Of all four markers, *Av4143* and *cytB* presented the highest sensitivity (72.7% and 87.0%) and specificity (87.5% and 82.4%) combination. Cross reactivity of *Av4143, cytb* and *ND5* was mainly associated with dog and pig fecal samples. Additionally, Av4143 also cross-reacted with one cat sample. The *LA35* marker was only able to identify 23.1% (3/13) known avian target samples, yet it did not react with any non-target sample (100% specificity).

**Table 3:** Internal validity metrics of all microbial source tracking markers tested in this study.

Target specie	Microbial source tracking marker	Including fecal samples from all species					Including fecal samples from contextually relevant species*				
		Sensitivity (%)	Specificity (%)	PPV (%)	NPV (%)	Accuracy (%)	Sensitivity (%)	Specificity (%)	PPV (%)	NPV (%)	Accuracy (%)
Domestic birds and waterfowl	*Av4143*	72.7	87.5	75.0	86.2	82.5	95.7	81.8	73.3	97.3	86.6
Chickens	*LA35*	23.1	100.0	100.0	89.4	89.7	23.1	100.0	100.0	84.4	85.1
Chickens and ducks	*cytB*	87.0	82.4	60.6	95.3	83.5	87.0	79.5	69.0	92.1	82.1
Chickens and ducks	*ND5*	69.6	75.7	47.1	88.9	74.2	69.6	70.5	55.2	81.6	70.1
Pigs	*Pig2bac*	100.0	88.5	50.0	100.0	89.7	100.0	98.2	90.9	100.0	98.5
Dogs	*Bactcan*	100.0	47.4	19.6	100.0	53.4	100.0	58.3	33.3	100.0	69.2
Humans	*Bachum*	80.0	66.2	50.0	88.7	70.3	80.0	62.5	57.1	83.3	69.2
Humans	*HF183-Taqman*	76.7	67.6	50.0	87.3	70.3	76.7	68.8	60.5	82.5	71.8

Key: (*) Contextually Relevant Species = Humans, dogs, cats, rats, chickens, ducks, pigs, parrots. Sensitivity = (True Positive / True Positive + False Negative); Specificity = (True Negative / True Negative + False Positive); Positive Predictive Value (PPV) = (True Positive / True Positive + False Positive); Negative Predictive Value (NPV) = (True Negative / True Negative + False Negative); Accuracy = (True Positive + True Negative / True Positive + True Negative + False Positive + False Negative).

Legend: Performance characteristics for 8 microbial source tracking markers to quantify feces of chickens, pigs, dogs, and humans demonstrate excellent performance of pig Pig2Bac MST marker, and avian marker Av4143. Dog and human markers demonstrate moderate performance.

**Table 4:** Sample-specific results of all fecal samples from Iquitos, Peru, tested with eight microbial source tracking markers.

Source	Microbial source tracking markers															
	*Av4143 (avian)*		*LA35 (avian)*		*CytB (avian)*		*ND5 (avian)*		*pig2bac (swine)*		*bactcan (canine)*		*bachum (human)*		*HF183 (human)*	
	Target	Non-Target	Target	Non-Target	Target	Non-Target	Target	Non-Target	Target	Non-Target	Target	Non-Target	Target	Non-Target	Target	Non-Target
Chicken	13/13	–	3/13	–	12/13	–	12/13	–	–	0/13	–	6/10	–	5/13	–	5/13
Duck	9/10	–	–	0/10	5/10	–	4/10	–	–	0/10	–	4/6	–	3/8	–	4/8
Pidgeon	2/10	–	–	0/10	–	4/10	–	4/10	–	0/10	–	2/10	–	0/3	–	0/3
Parrot	–	0/2	–	0/2	–	0/2	–	1/2	–	0/2	–	NA	–	0/1	–	0/1
Children	–	0/10	–	0/10	–	2/10	–	3/10	–	0/10	–	0/10	11/15	–	11/15	–
Adults	–	NA	–	NA	–	NA	–	NA	–	NA	–	NA	13/15	–	12/15	–
Guinea pig	–	0/5	–	0/5	–	0/5	–	1/5	–	0/5	–	5/5	–	0/5	–	0/5
Rat	–	1/10	–	0/10	–	0/10	–	0/10	–	0/10	–	0/10	–	5/10	–	4/10
Goat	–	0/10	–	0/10	–	0/10	–	0/10	–	9/10	–	9/10	–	5/10	–	6/10
Buffalo	–	0/5	–	0/5	–	0/5	–	0/5	–	0/5	–	5/5	–	1/5	–	2/5
Pig	–	3/10	–	0/10	–	4/10	–	4/10	10/10	–	–	10/10	–	2/5	–	2/5
Dog	–	3/10	–	0/10	–	3/10	–	5/10	–	1/10	10/10	–	–	2/9	–	0/9
Cat	–	1/2	–	0/2	–	0/2	–	0/2	–	0/2	–	0/2	–	1/2	–	0/2
Total target positive samples	24/33	–	3/13	–	20/23	–	16/23	–	10/10	–	10/10	–	24/30	–	23/30	–
Total non-target positive samples	–	8/64	–	0/84	–	13/74	–	18/74	–	10/87	–	41/78	–	24/71	–	24/71

Legend: Sample specific results of each microbial source tracking marker, showing all eight markers, except LA35, are able to detect the majority of target samples. Dog marker (Bactcan), and human markers (*BacHum* and HF-Taqman) show a high proportion of cross reactivity with non-target samples.

Of the mammalian markers, *Pig2Bac* showed the best performance parameters, (100.0% sensitivity and 88.5% specificity), detecting 100% of target samples and only 11.5% (11/87) of non-target samples. As specified in Table 3, cross-reactivity of *Pig2Bac* was mainly associated with goat fecal samples, a species that is occasionally kept as pets or as food production animals in this setting. Both human markers targeted a *Bacteroides* sp. gene segment, with similar sensitivity and specificity values. *BacHum* detected 80.0% (24/30) of target samples and *HF183-Taqman* 76.7% (23/30). Both markers detected 33.8% of non-target samples, with highest cross-reactivity associated with goats and rats. *BactCan*, the dog-associated marker showed very low specificity, and cross-reacted with pig and goat samples.

When excluding the results from goats, guinea-pigs, buffalos and pigeons, given their lack of representativeness in this community, the sensitivity of *Av4143* increased significantly (72.7% vs. 95.7%). Sensitivities for the rest of the markers did not change. Specificities increased for *Pig2Bac* (from 88.5% to 98.2%), *BactCan* (from 47.4% to 58.3%) and *HF183-Taqman* (from 67.6% to 68.8%), yet decreased for *Av4143* (from 87.5% to 81.8%), *cytb* (from 82.4% to 79.5%), *ND5* (from 75.7% to 70.5%) and *BacHum* (66.2% to 62.5%) (Table 3).

**Fig. 2:**
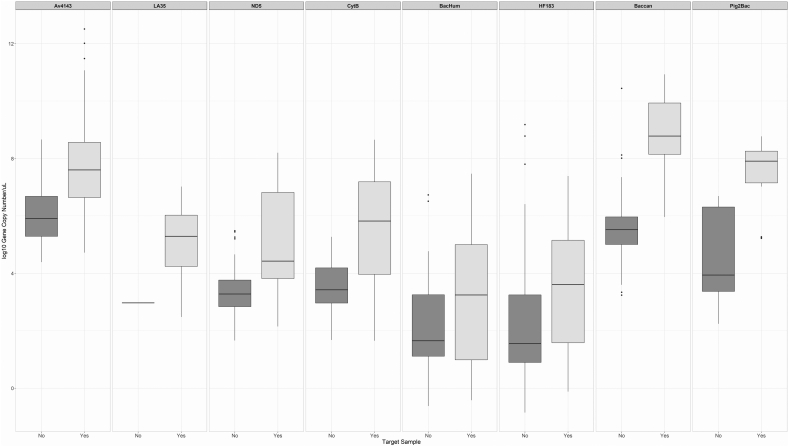
Quantitative analysis of (log10) gene copy number quantities of the eight MST markers validated in target and non-target fecal samples. Key: Target samples refer to fecal samples of animal species for which the microbial source tracking was developed. Non-target samples refer to fecal samples of animal species for which the microbial source tracking marker was not develop. Legend: All microbial source tracking markers, except LA35, are able to detect statistically significant higher gene copy numbers among target samples in comparison to non-target samples.

We did a quantitative assessment of the log(Humphrey et al., 2019) transformed gene copy numbers detected by each marker in both target and non-target species. Data is displayed in Fig. 2 and Supplementary Fig. 1. All markers (except of *LA35*) were able to detect higher gene copy numbers in target samples in comparison to non-target samples. Finally, avian markers *ND5* and *cytB* had a statistically-significant (Bonferroni adjusted 0.05 significance level) positive correlation (Pearson's correlation coefficient, 0.949), as well as *cytB* and *Av4143* (Pearson's correlation coefficient, 0.508). Human markers *HF183-Taqman* and *BacHum* also showed a statistically-significant positive correlation (Pearson's correlation coefficient, 0.786). A pairwise correlation coefficient matrix is presented in Supplementary Table 3.

## 4 Discussion

This study validated eight microbial source tracking markers for the proposed principal sources of fecal contamination for communities in the Peruvian Amazon. This work informs use of these markers to understand the relative contribution of human and animal fecal contamination in household environments and further demonstrates that the setting in which fecal samples was collected does influence the MST marker performance.

Various MST markers targeting human feces have developed for multiple purposes. Bacteroidales genetic markers are particularly common, yet prior work demonstrates that the performance of these markers may vary widely. In addition, few markers have been validated in low-resource developing areas of the world. In this study we validated the *BacHum* and *HF183-Taqman*, both of which have also been evaluated in Thailand (100 composite samples), Singapore (35 human sewage samples), Nepal (10 composite samples composed of 10 samples each), India (35 human samples) and Kenya (12 human samples) (Somnark et al., 2018; Nshimyimana et al., 2017; Malla et al., 2018; Odagiri et al., 2015). In this study, *BacHum* showed a sensitivity of 80% and specificity of 66%. Sensitivity parameters in these other countries have not been consistent, with values ranging from 95 to 100% in Thailand and Nepal, to 65% in Singapore, 50% in India and 18% in Kenya (Somnark et al., 2018; Nshimyimana et al., 2017; Malla et al., 2018; Odagiri et al., 2015; Jenkins et al., 2009). Specificity parameters in these same countries ranges from 54% (Thailand), 77% (Nepal), 78% (India), 91% (Singapore) and 100% (Kenya) (Somnark et al., 2018; Nshimyimana et al., 2017; Malla et al., 2018; Odagiri et al., 2015; Jenkins et al., 2009).

*HF183* has been developed for both SYBR green and Taqman technologies. In this study, *HF183-Taqman* showed a sensitivity of 77% and specificity of 67%. As with *BacHum*, internal validity metrics are not consistent across studies, with values ranging from 29% in India, 60% in Singapore and 84–100% in Thailand and Nepal. Specificity values are inconsistent, ranging from 70% in Nepal, 80% in India, 91% in Singapore and 77–100% in Thailand (Somnark et al., 2018; Nshimyimana et al., 2017; Malla et al., 2018; Odagiri et al., 2015). Validation studies done in Australia for both *HF183* and *BacHum* report 95–100% sensitivity and a specificity that ranges between 79% and 99% (Ahmed et al., 2009; Ahmed et al., 2008; Ahmed et al., 2012).

Reasons for the wide variation in parameters across studies could be multiple, such as differences in human microbiome, genetic variability of Bacteroides, and differences in the climate and local ecology which may indirectly affect microbial populations (Boehm et al., 2013). Age differences could also account for such differences; yet in this study, results from adults and children were not significantly different (data not shown). The need for a consistently well-performing human microbial source tracking marker is evident.

Several swine microbial source tracking markers have been developed, yet *Pig-2-Bac* has shown consistently strong performance across studies. In this study, *Pig-2-Bac* showed a sensitivity of 100% and was the most specific marker (88.5%), and it only cross-reacted with goats (a non-relevant animal in this context) and one dog. The reason for such high cross-reactivity with goats warrants further investigation in future studies. Similar results were evidenced in Thailand and China (Somnark et al., 2018; He et al., 2016), with lower specificity in Nepal (75%) (Malla et al., 2018). The *BactCan* dog marker showed very low specificity in this study, cross reacting with goats, pigs, chickens and guinea pigs. Similarly, in Nepal this same marker shows a specificity of 45%, yet in India and Singapore it was quite found to be high (97%) (Nshimyimana et al., 2017; Malla et al., 2018; Odagiri et al., 2015). Multi-species cohabitation is common in this setting, with a high frequency of coprophagy in both dogs, chickens and pigs. In this particular validation study, pigs were confined in a production facility and as a result, there is a low probability of ingestion of dog or any other animal fecal material. Dogs however, are seldom confined or fed pet kibble and scavenge extensively and are coprophagic and will scavenge intestines following household slaughter.

Few studies have employed avian MST markers for the attribution of fecal contamination relative to studies validating and implementing human and mammalian MST markers. *LA35* was developed in the United States for the detection of poultry litter, showing low sensitivity for poultry fecal samples, similarly to what this study determined (Weidhaas et al., 2010; Weidhaas and Lipscomb, 2013; Ryu et al., 2014; Weidhaas et al., 2011). *Av4143*, was developed in Israel and validated in a single study in China, with a sensitivity of 100% and specificity of 95%, with cross-reactivity with human and cow samples (Ohad et al., 2016; Vadde et al., 2019). In this study, *Av4143* cross-reacted with dogs, cats and pig fecal samples, and had a sensitivity of 72.7% when considering all fecal samples tested, and of 95.7% when considering only relevant animals for this community. The two mitochondrial avian markers, *cytB* and *ND5* have not been validated in other settings, besides China, where they were developed. There are other avian fecal markers that have been developed, such as those targeting a *Faecalibacterium* 16S rDNA gene (Green et al., 2012; Shen et al., 2013), as well as the other four markers developed concomitantly with Av4143 (Ohad et al., 2016). Finally, avian-GFD, a *Helicobacter* spp. gene segment specific for fecal samples from chickens and waterfowl species, has been validated and used in Bangladesh, Mozambique, the United States, Canada and New Zealand (Harris et al., 2016; Boehm et al., 2016; Green et al., 2012; Holcomb et al., 2020; Nguyen et al., 2018).

The validation of MST markers in Peruvian Amazon opens the potential to study animal fecal exposure to a species level within the household domain. This is of critical importance given that the burden of zoonotic enteric infections in these communities is high, with *Campylobacter* spp., ST-ETEC accounting for more than 10% of symptomatic enteric disease in children under the age of two (Platts-Mills et al., 2015; Amour et al., 2016). The indoor environments of households in these communities have been shown to contain high quantities of fecal material, and its built environment (such as the flooring material) is associated with the burden of *Escherichia coli* in household surfaces (Exum et al., 2016). Identifying the source of fecal contamination within the indoor-household environment has the potential to restructure water, sanitation and hygiene research and interventions with the ultimate goal of reducing enteric disease and associated developmental consequences. This validation study is the first step towards achieving this goal.

The use of microbial source tracking markers to detect and quantify the source of fecal contamination in this setting has inherent limitations. Multi-species cohabitation alongside humans is common. As a result, ingestion of another species by a second relevant species, or the ingestion of feces of other species is not infrequent and likely is a factor that diminished the specificity of the markers in this field setting. For instance, it is common to observe dogs, which are never confined, consuming matter on infant diapers or avian viscera. Although this may be seen as a limitation, it is a reflection of the real world performance in transmission studies in most regions where diarrhea is highly endemic. Future studies should track co-habitation and *peri* domestic livestock husbandry in attempt to elucidate the reason for highly variable false-positivity rates in order to improve source attribution. This issue could potentially have large implications for MST performance in low-income settings within One Health studies.

## 5 Conclusion

This study provides the first performance evaluation of six bacteria-associated and two mitochondria-associated microbial source tracking markers for the attribution of fecal contamination to a species-specific level in a low-resource tropical community of the Amazon. Based on their performance parameters, *Pig-2-Bac, Av4143, cytB* and *HF183*-Taqman have the highest potential to be implemented in this community setting. These microbial source tracking markers are an attractive tool for future studies that aim to elucidate the source of fecal exposure within household environments with high burden of fecal exposure given that they have potential to uncover new disease control interventions tailored to the fecal exposure landscape and animal husbandry practices of low-resource communities in developing world.

## CRediT authorship contribution statement

**Francesca Schiaffino:** Conceptualization, Methodology, Formal analysis, Investigation, Writing - original draft, Visualization. **Nora Pisanic:** Conceptualization, Methodology, Writing - original draft, Visualization. **Josh M. Colston:** Formal analysis, Writing - original draft, Visualization. **Dixner Rengifo:** Formal analysis, Methodology, Investigation. **Maribel Paredes Olortegui:** Conceptualization, Methodology, Investigation, Supervision. **Valentino Shapiama:** Formal analysis, Methodology, Investigation. **Pablo Peñataro Yori:** Conceptualization, Methodology, Investigation. **Christopher D. Heaney:** Conceptualization, Writing - review & editing. **Meghan F. Davis:** Conceptualization, Methodology, Writing - original draft, Visualization. **Margaret N. Kosek:** Conceptualization, Methodology, Formal analysis, Investigation, Writing - original draft, Supervision, Funding acquisition.

## Declaration of competing interest

The authors declare that they have no known competing financial interests or personal relationships that could have appeared to influence the work reported in this paper.

## Appendix A Supplementary data

Supplementary material Image 1
