# Supplementary material for: Validation of microbial source tracking markers for the attribution of fecal contamination in indoor-household environments of the Peruvian Amazon
Source: Sci Total Environ. 2020 Nov 15;743:140531. doi: 10.1016/j.scitotenv.2020.140531 (PMC7511695; doi:10.1016/j.scitotenv.2020.140531)

Supplementary Material

Supplementary Table 1: Sex and age of human fecal samples used in the validation process

Adults	Sex	Age (years)	Children	Sex	Age (years)
Sample 1	Male	46	Sample 1	Male	3
Sample 2	Male	22	Sample 2	Female	4
Sample 3	Female	36	Sample 3	Male	3
Sample 4	Male	51	Sample 4	Male	3
Sample 5	Female	36	Sample 5	Female	5
Sample 6	Female	49	Sample 6	Female	2
Sample 7	Male	48	Sample 7	Male	4
Sample 8	Female	37	Sample 8	Male	3
Sample 9	Male	37	Sample 9	Female	5
Sample 10	Female	35	Sample 10	Female	3
Sample 11	Female	36	Sample 11	Female	3
Sample 12	Male	32	Sample 12	Female	1
Sample 13	Male	34	Sample 13	Female	2
Sample 14	Female	18	Sample 14	Male	5
Sample 15	Female	33	Sample 15	Male	3

Supplementary Table 2: Gblock sequences used for the development of the eight standard curves.

MST	Final Control Sequence for gBlock
LA35_control	tgcatgatctacgtgcgtcacatgcagtacACCGGATACGACCATCTGCCGCATGGCGGGTGGTGGAAAGTTTTTCGATTGGGGATGGG<br>CTCGCGGCCTATCAGTTTGTTGGTGGGGTAATGGCCTACCAAGGCGACGACGGGTAGCCGGCCTGAGAGGGCGACCG<br>GCCACACTGGGACTGAGACACGGCCCAGACTCCTACGGGAGGCAGCAGTGGGGAATATTGCACAATGGGGGAAACC<br>CTGATGCAGCGACGCAGCGTGCGGGATGACGGCCTTCGGGTTGTAAACCGCTTTCAGCAGGGAAGAAGCCTTCGGGT<br>GACGGTACCTGCAGAAGAAGTACCGGCTAACTACGTGCCAGCAGCCGCGGTAATACGTAGGGTACGAGCGTTGTCCG<br>GAATTATTGGGCGTAAAGAGCTCGTAGGTGGTTGGTCACGTCTGCTGTGGAAACGCAACGCTTAACGTTGCGCGGGC<br>AGTGGGTACGGGCTGACTAGAGTGCAGTAGGGGAGTCTGGAATTCCTGGTGTAGCGGTGAAATGCGCAGATATCAGG<br>AGGAACACCGGTGGCGAAGGCGGGACTCTGGGCTGTGACTGACACTGGGGAcactagctcagattcagtagaccgctgttg
ND-5_control	tgcatgatctacgtgcgtcacatgcagtacACCTCCCCCAACTAGCCTTCCTCCACATCTCAACCCATGCCTTCTTTAAAGCTATATTATT<br>CCTATGCTCCGGCCTAATTATCCACAGCCTCAATGGAGAACAAGACATCCGCAAAATAGGATGTCTACAAAAAACCC<br>TCCCAATAACCACCTCCTGCCTAACCATTGGCAAcactagctcagattcagtagaccgctgttg
cytbf_control	tgcatgatctacgtgcgtcacatgcagtacAAATCCCACCCCCTACTAAAAATAATTAACAACTCCCTAATCGACCTCCCAGCCCCATCC<br>AACATCTCTGCTTGATGAAATTTCGGCTCCCTATTAGCAGTCTGCCTCATGACCCAAATCCTCACCGGCCTACTACTAG<br>CCATGCACTACACAGCAGACACATCCCTAGCCTTCTCCTCCGTAGCCCACACTTGCCGGAACGTACAATACGGCTGAC<br>TCATCCGGAATCTCCACGCAAACGGCGCCTCATTCTTCTTCATCTGcactagctcagattcagtagaccgctgttg
Av4143_control	tgcatgatctacgtgcgtcacatgcagtacTGCAAGTCGAACGAGGATTTCTTACACTGAGTGCTTGCACTCACCGTAAGAAATTCGAGT<br>GGCGGACGGGTGAGTAACACGTGGGTAACCTGCCCAAAAGAAGGGGATAACATTTGGAAACAAATGCTAATACCGT<br>ATAACCATGATGACCGCATGGTCATTATGTAAAAGGTGGTTTTGCTATCGCTTTTGGATGGACCCGCGGCGTATTAAC<br>TAGTTGGTAGGGTAACGGCCTACCAAGGTGAcactagctcagattcagtagaccgctgttg
BactCan545f1_control	tgcatgatctacgtgcgtcacatgcagtacGGAGCGCAGACGGGTTTTTAAGTCAGCTGTGAAAGTTTGGGGCTCAACCTTAAAATTGC<br>AGTTGATACTGGAGACCTTGAGTGCAGTTGAGGCAGGCGGAGTTCGTGGTGTAGCGGTGAAATGCTTAGATATCACG<br>AAGAACTCCGATTGAcgctgtgtcgtaacTAGATATCACGAGGAACTCCGATTcactagctcagattcagtagaccgctgttg
Pig-2-Bac41F_control	tgcatgatctacgtgcgtcacatgcagtacGCATGAATTTAGCTTGCTAAATTTGATGGCGACCGGCGCACGGGTGAGTAACGCGTATC<br>CAACCTTCCCTTATCCACGGGATAGCCCGTCGAAAGGCGGATTAATACCGTATGAGGTcactagctcagattcagtagaccgctgttg
*HF183f*_control	tgcatgatctacgtgcgtcacatgcagtacCGTAGGAGTTTGGACCGTGTCTCAGTTCCAATGTGGGGGACCTTCCTCTCAGAACCCCTA<br>TCCATCGTTGACTAGGTGGGCCGTTACCCCGCCTACTATCTAATGGAACGCATCCCCATCGTCTACCGGAAAATACCT<br>TTAATCATGCGGACATGTGAACTCATGATcactagctcagattcagtagaccgctgttg
BacHum	ACGGGTGAGTAACACGTATCCAACCTGCCGTCTACTCTTGGACAGCCTTCTGAAAGGAAGATTAATCCAGGATGGCAT<br>CATGAGTTCACATGTCCGCATGATTAAGGTATTCCGGTAGACGATGGGGATGCGTTCCATTAGATAGTAGGCGGGGTA<br>ACGGCCCACCTAGTCTTCGATGGATAGGGGTTCTGAGAGGAAGGTCCCCCACATTGGAACTGAGACACGGTCCAA

Supplementary Table 3: Correlation matrix with pairwise pearson's correlation coefficients of the log(10) gene copy number per microliter of the eight microbial source tracking markers among all fecal samples tested.

	Av4143	*ND5*	*CytB*	*BacHum*	*HF183*	*Bactcan*	*Pig2Bac*
Av4143	1.0000						
ND5	0.5176	1.0000					
CytB	0.5083*	0.9492*	1.0000				
BacHum	0.1294	-0.2385	0.3554	1.0000			
HF183	-0.0187	0.0399	-0.0513	0.7855*	1.0000		
Bactcan	-0.1632	0.2950	0.0894	-0.2258	-0.3780	1.0000	
Pig2Bac	0.3473	-0.7071	0.1253	0.1175	-0.0751	-0.1594	1.0000

(*)

Bonferroni Adjusted Significance level of 0.05.

Supplementary Figure 1: Quantitative (log(10) gene copy number/uL) results of fecal samples of distinct animal species tested with eight microbial source tracking markers

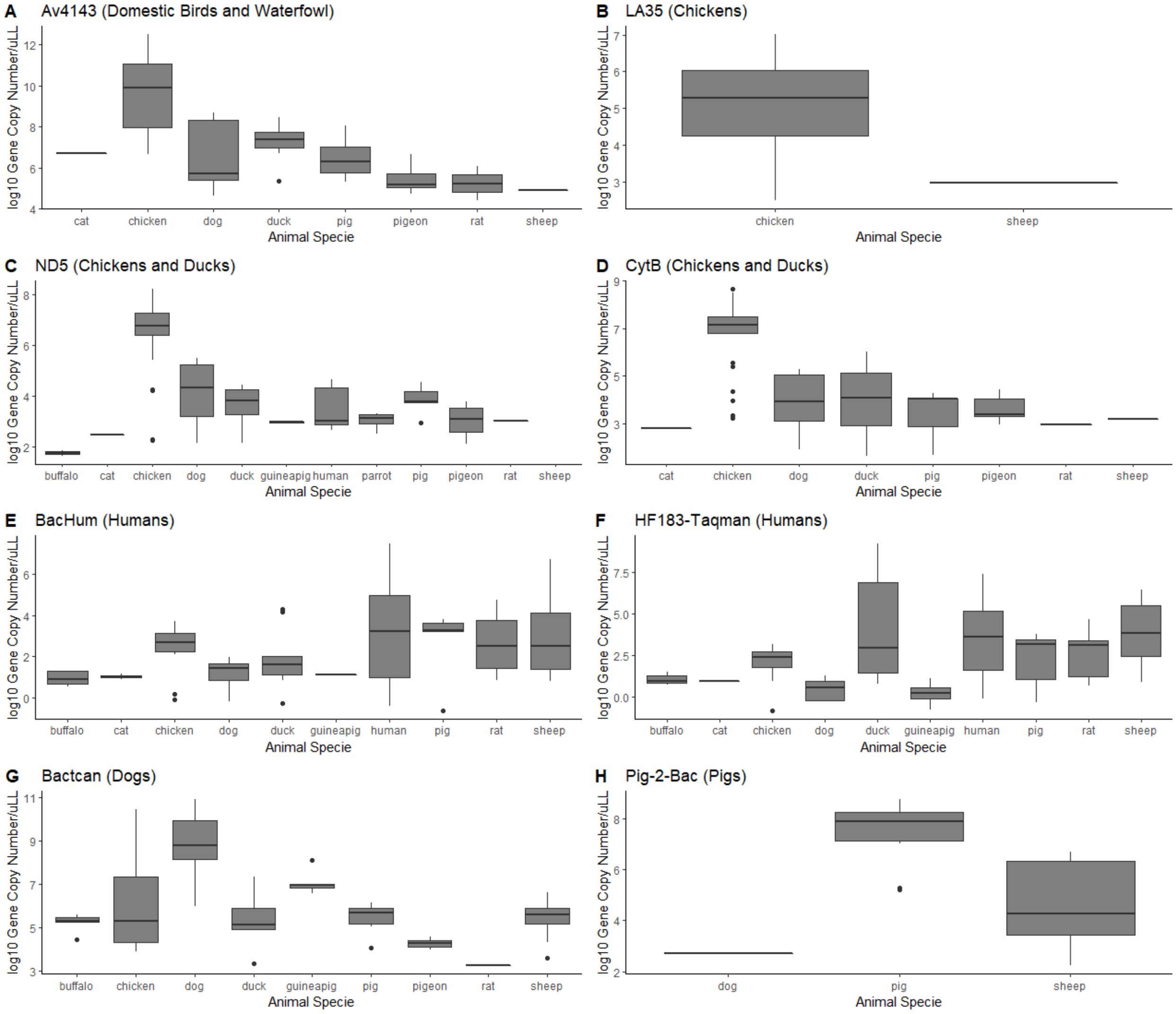

Supplement: Supplementary file 1 — Supplementary material [file mmc1.pdf]
